# Quality and transparency of reporting derivation and validation prognostic studies of recurrent stroke in patients with TIA and minor stroke: a systematic review

**DOI:** 10.1186/s41512-022-00123-z

**Published:** 2022-05-19

**Authors:** Kasim E. Abdulaziz, Jeffrey J. Perry, Krishan Yadav, Dar Dowlatshahi, Ian G. Stiell, George A. Wells, Monica Taljaard

**Affiliations:** 1 Clinical Epidemiology Program, Ottawa Hospital Research Institute, Ottawa, Ontario Canada; 2 School of Epidemiology and Public Health, Faculty of Medicine, University of Ottawa, Ottawa, Ontario Canada; 3 Department of Emergency Medicine, University of Ottawa, Ottawa, Ontario Canada; 4 Department of Medicine (Neurology), University of Ottawa, Ottawa Hospital Research Institute, Ottawa, Ontario Canada; 5 Cardiovascular Research Methods Centre, University of Ottawa Heart Institute, Ottawa, Ontario Canada

**Keywords:** Prediction models, Prediction rules, Clinical decision rules, Risk scores, Recurrent stroke, Transient ischemic attack, Cerebral ischemia, TRIPOD, CHARMS, Reporting quality

## Abstract

**Background:**

Clinical prediction models/scores help clinicians make optimal evidence-based decisions when caring for their patients. To critically appraise such prediction models for use in a clinical setting, essential information on the derivation and validation of the models needs to be transparently reported. In this systematic review, we assessed the quality of reporting of derivation and validation studies of prediction models for the prognosis of recurrent stroke in patients with transient ischemic attack or minor stroke.

**Methods:**

MEDLINE and EMBASE databases were searched up to February 04, 2020. Studies reporting development or validation of multivariable prognostic models predicting recurrent stroke within 90 days in patients with TIA or minor stroke were included. Included studies were appraised for reporting quality and conduct using a select list of items from the Transparent Reporting of a Multivariable Prediction Model for Individual Prognosis or Diagnosis (TRIPOD) Statement.

**Results:**

After screening 7026 articles, 60 eligible articles were retained, consisting of 100 derivation and validation studies of 27 unique prediction models. Four models were newly derived while 23 were developed by validating and updating existing models. Of the 60 articles, 15 (25%) reported an informative title. Among the 100 derivation and validation studies, few reported whether assessment of the outcome (24%) and predictors (12%) was blinded. Similarly, sample size justifications (49%), description of methods for handling missing data (16.1%), and model calibration (5%) were seldom reported. Among the 96 validation studies, 17 (17.7%) clearly reported on similarity (in terms of setting, eligibility criteria, predictors, and outcomes) between the validation and the derivation datasets. Items with the highest prevalence of adherence were the source of data (99%), eligibility criteria (93%), measures of discrimination (81%) and study setting (65%).

**Conclusions:**

The majority of derivation and validation studies for the prognosis of recurrent stroke in TIA and minor stroke patients suffer from poor reporting quality. We recommend that all prediction model derivation and validation studies follow the TRIPOD statement to improve transparency and promote uptake of more reliable prediction models in practice.

**Trial registration:**

The protocol for this review was registered with PROSPERO (Registration number CRD42020201130).

**Supplementary Information:**

The online version contains supplementary material available at 10.1186/s41512-022-00123-z.

## Background

Clinical prediction models (also called clinical prediction rules, clinical prediction scores, clinical decision rules, or prognostic models) aid clinicians in making diagnostic and therapeutic decisions at the bedside and reduce inefficient provision of resources when presented and applied appropriately [1–3]. Such tools are commonly used by clinicians, and especially in the emergency department, to identify patients at high risk of stroke as it is not practical to treat everyone due to limited resources. Transient ischemic attack (TIA, i.e. a cerebral ischemia without lasting symptoms) and minor stroke carry a serious risk of subsequent stroke or death shortly after diagnosis, and thus represent an opportunity for stroke prevention [4].

To maximize the accuracy and clinical utility of clinical prediction models, they need to go through at least two consecutive phases: derivation including internal validation and external validation (evaluation to check accuracy in an independent population and setting) [2, 5–9]. Numerous methodological standards and guides have been developed for clinical prediction modelling [3, 6, 10–14]. Unfortunately, several systematic reviews have indicated shortcomings in methodological quality of many existing prediction studies [14, 15]. In addition to methodological standards for the development and validation of clinical prediction models, appraisal guidelines such as the CHecklist for critical Appraisal and data extraction for systematic Reviews of prediction Modelling Studies (CHARMS) [16] and the Prediction model Risk Of Bias ASsessment Tool (PROBAST) [17] have been developed for data extraction and critical appraisal and assessment of risk of bias in modelling studies. To use such appraisal guides to their full extent, essential items need to be reported in the paper deriving or validating a prediction model. The strengths and weaknesses of a prediction model study can only be revealed with full and transparent reporting to enable its interpretation and usefulness and enhance the uptake and implementation of validated models for use in clinical settings [18]. Complete and transparent reporting also support future prediction model studies by allowing researchers to validate and compare existing prediction models [18]. For this reason, reporting guidelines such as the Transparent Reporting of a Multivariable Prediction Model for Individual Prognosis or Diagnosis (TRIPOD) have been developed for studies developing, validating, or updating a prediction model [19].

Several clinical prediction models exist for the prognosis of stroke in patients with TIA and minor stroke. To critically appraise their methodological quality and make recommendations about future updates and/or adoption in clinical practice, better reporting quality of prediction models is essential. Although several systematic reviews have been conducted of the quality of reporting of prediction model studies in various other clinical domains [15, 20–28], to our knowledge, there have been no reviews of the reporting quality of prognostic models for stroke. Thus, we aimed to identify existing clinical prediction models and assess their reporting quality using the recommendations in the TRIPOD statement.

The overall goal of this study is to critically appraise existing derivation and validation studies of prediction models, in terms of reporting quality, for the prognosis of recurrent stroke within 90 days in patients with TIA or minor stroke. The specific objectives are:

1. To identify and characterize derivation and validation studies of existing multivariable clinical prediction models in the literature for the prognosis of recurrent stroke within 90 days in patients with TIA or minor stroke;
2. To characterize the quality of reporting of a select list of essential items for both the derivations and validations.

## Methods

**Fig. 1:**
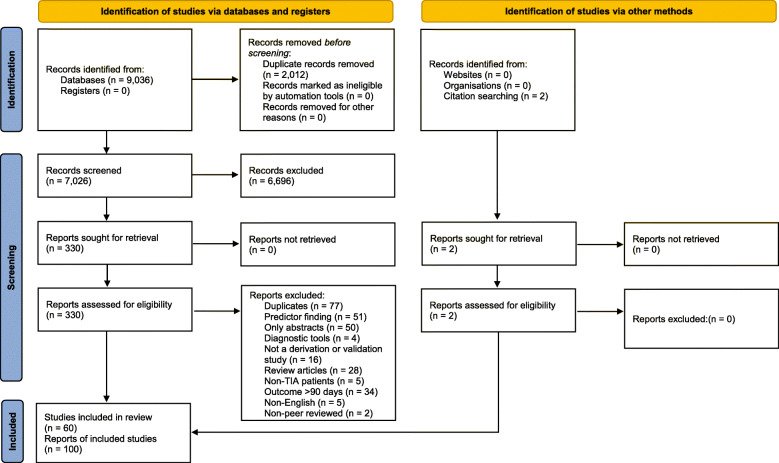
Study selection adapted from PRISMA [29]

This review followed the Preferred Reporting Items for Systematic Reviews and Meta-Analysis (PRISMA) statement (See Fig. 1 and Additional file 1) [29]. To help us guide the framing of the review aim, search strategy, and study inclusion and exclusion criteria, we used key items from the TRIPOD and the CHARMS Checklist, presented in additional files (See Supplementary Table 1, Additional file 2).

### Search strategy

We conducted comprehensive electronic searches to identify all published studies of clinical prediction models for the prognosis of recurrent stroke within 90 days in patients with TIA or minor stroke.

We searched the Ovid interface, as well as Medline and Embase databases using a strategy that included the National Library of Medicine’s Medical Subject Headings (MeSH) and non-MeSH keywords up to February 04, 2020. The search strategy used TIA and stroke search syntax and used established search filters for prediction models [30–33]. Due to the low quality of reporting of prediction modelling studies, we further modified the search filter by including additional and more specific search terms to be more comprehensive in identifying relevant studies. We developed the search strategy with the help of an experienced medical librarian. It was later validated by a second medical librarian in accordance with the Peer Review of Electronic Search Strategies (PRESS) guidelines [34]. A copy of the search strategy can be found in additional files (See Additional file 3). We also used Google Scholar to search and find any additional articles by searching for relevant keywords in the Google Scholar search engine. In addition, we searched the citations from the included studies for additional eligible studies of clinical prediction models.

### Inclusion and exclusion criteria

This systematic review focused on studies of any design that developed or validated multivariable prediction models or scoring rules for the prognosis of recurrent stroke within 90 days for patients diagnosed with TIA or minor stroke.

We considered a clinical prediction model to be any tool that combined at least two predictors to estimate a probability or score for the outcome of stroke within 90 days. We excluded studies that investigated a single predictor, test, or marker. We also excluded studies that investigated only causality between one or more variables and an outcome, and predictor finding studies, i.e. studies which aim to explore which predictors, out of a number of candidate predictors, are independently associated with a diagnostic or prognostic outcome rather than deriving or validating a prediction model [14]. We excluded five studies in languages other than English. We did not apply publication date restrictions.

### Screening

After retrieving the potentially relevant articles from the search, we imported the records into Covidence (https://www.covidence.org). We removed duplicates and two reviewers (KEA and KY) independently screened the title, abstract, and keywords. Prior to embarking on screening, the two reviewers screened a sample of records as part of a training and calibration exercise, and the screening criteria were clarified where necessary.

We retrieved the full text of the articles that were considered potentially relevant by at least one of the reviewers in the title or abstract screen. One of the reviewers then reviewed the full text of each of the articles to determine eligibility. We excluded articles for which we could not obtain full text.

### Data extraction

One reviewer (KEA) extracted data from each included study using an extraction form that was specifically designed and pilot tested for the review. We extracted data separately for each derivation or validation cohort (hereafter called cohort) in a publication.

The data extraction form was based on selected items from the TRIPOD and CHARMS checklists for reporting and critical appraisal of prognostic model studies. We focused on items that are essential for the appraisal of derivation and validation studies. Although we initially set out to assess both reporting quality and quality of methodological conduct, we ultimately decided to focus on quality of reporting as our appraisal of methodological conduct was hampered by a lack of clarity in the reporting. For feasibility reasons and to keep the project manageable, we did not assess adherence to all the TRIPOD items. We extracted information pertaining to items 1, 3 through 9, 13, 14, 16, and 22 of the TRIPOD statement, covering the title, background and rationale, methods, and results along with information about funding that was thought to be particularly relevant to the field; we did not extract information about items 2, 10, 11, 15, 17 through 21 covering the abstract, specifics of statistical analysis methods, model specification, discussion, and other information (supplemental information) sections. Some TRIPOD items are applicable to both derivation and validation studies, while others are applicable to validation studies only. We distinguished between validation studies without updating and validation studies with updating (i.e. studies developing an updated model based on an existing model). For validation studies with updating, we also extracted whether the update was performed in accordance with suggested methodology by Su and colleagues [35]. In particular, we extracted whether the order of performing the update was as follows: (a) recalibrating the intercept only, (b) recalibrating the intercept and adjusting the other regression coefficients by a common factor, (c) category b plus extra adjustment of a subset of the existing coefficients to a different strength, (d) category c plus adding new predictors, (e) re-estimating all of the original regression coefficients, and (f) category e plus adding new additional predictors [35]. This order of performing the update was recommended so that authors consider less extensive update methods prior to considering more extensive revisions. For example, it might suffice to recalibrate the intercept only to improve the performance of the model in the setting it is being validated in.

A list of extracted items can be found in additional files (See Supplementary Table 2, Additional file 2).

As this was a study on quality of reporting, we did not perform risk of bias assessment on individual studies. We have registered the protocol for this study with PROSPERO (Registration number CRD42020201130) prior to extracting the data.

### Analysis

We summarized and presented the results of this systematic review using descriptive statistics (numbers and percentages). We classified each item as adherent, not adherent, or unclear or not reported. We combined a few studies classified as non-adherent with unclear or not reported to create a combined category of “Non-adherence, unclear, or not reported”. In other words, we consider not reporting or unclear reporting as a form of non-adherence.

## Results

### Search results

Our database search identified 9036 articles. After removal of duplicates, 7026 titles and abstracts were screened for eligibility. After title and abstract screening, 6696 records were excluded leaving 330 full-text articles for eligibility assessment. Two additional articles were later identified by hand searching the reference lists. A total of 60 articles ultimately met our inclusion criteria (Fig. 1). Reasons for exclusion are outlined in Fig. 1.

### Study characteristics

A total of 100 derivation and validation studies were performed on 27 prediction models within 60 published articles. Among the 100 derivation and validation studies, 27 were classified as prediction model development (either anew or by model updating) while the remaining 73 were classified as validation. Of the 27 prediction models, four were newly developed (i.e. not based on existing models) while 23 were developed based on existing models (i.e. validation with model updating). All 60 articles were published between 2000 and 2020. The studies were conducted in 18 different countries, mostly the UK (8 or 13.3%) and China (8 or 13.3%) followed by the USA (7 or 11.7%). Countries with three or fewer studies were grouped together under the other category and included the following countries: Austria, Bulgaria, Canada, Germany, Greece, Italy, Japan, Norway, Singapore, Spain, Sweden, Switzerland, and Turkey. The country of the study was not reported for 4 (6.7%) of the studies.

**Fig. 2:**
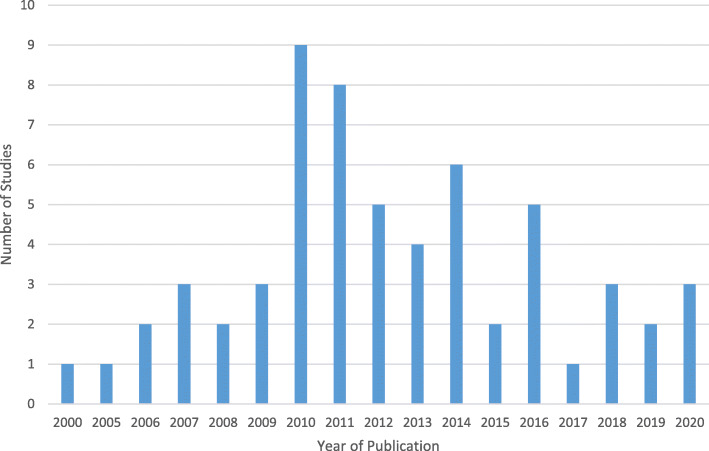
Distribution of included studies by year of publication

A list of the included studies can be found in the additional files (See Additional file 4). Distribution of included studies by year of publication is presented in Fig. 2.

### Reporting of items applicable at the article level

Results of reporting of items applicable at the article level are presented in Table 1. According to the TRIPOD statement, an informative study title entails identifying the study as derivation or validation of a multivariable prediction model, the target population, and the outcome to be predicted (TRIPOD item 1). Fifteen out of 60 studies (25.0%) adhered to the title recommendations. The source and role of funders (TRIPOD item 22) was provided by 14 (23.3%) of the 60 published articles.

**Table 1:** Reporting of items applicable at the article level

Characteristic	Number (%) ***N*** = 60
**Clear reporting in the title of the study as derivation or validation of a multivariable prediction model, the target population, and the outcome to be predicted**	
Yes	15 (25.0)
**Country of study**	
UK	8 (13.3)
China	8 (13.3)
USA	7 (11.7)
Iran	5 (8.3)
Australia	4 (6.7)
Others	24 (40.0)
Unclear or not reported	4 (6.7)
**Source and role of funders provided**	
Yes	14 (23.3)

### Reporting of essential items common to both derivation and validation studies

Results of reporting of essential items common to derivation and external validation studies are presented in Table 2.

**Table 2:** Reporting of essential items applicable to both derivation and validation studies

Characteristic	Number (%) ***N*** = 100
**General information**	
**Rationale provided for the derivation or validation of the model**	
Yes	66 (66.0)
**Source of data**	
Prospective cohort	60 (60.0)
Retrospective cohort—registry data	17 (17.0)
Retrospective cohort—data from past studies	12 (12.0)
Retrospective cohort—chart review	10 (10.0)
Unclear or not reported	1 (1.0)
**Inclusion criteria** (definition used for selection)	
TIA—Classical time-based definition	92 (92.0)
TIA—Tissue-based definition	5 (5.0)
Minor Stroke—Classical time-based definition	8 (8.0)
Minor Stroke—Tissue-based definition	1 (1.0)
Unclear or not reported	7 (7.0)
**Time-based versus tissue-based inclusion criteria**	
Time-based TIA/minor stroke	88 (88.0)
Tissue-based TIA/minor stroke	5 (5.0)
Unclear or not reported	7 (7.0)
**TIA versus minor stroke inclusion criteria**	
TIA only	85 (85.0)
Both TIA and minor stroke	8 (8.0)
Unclear or not reported	7 (7.0)
**Definition used for outcome of stroke**	
Clinical definition	73 (73.0)
Tissue-based definition	7 (7.0)
Unclear or not reported	20 (20.0)
**Time of outcome prediction**	
2-day	28 (28.0)
3-day	2 (2.0)
7-day	66 (66.0)
14-day	2 (2.0)
28-day	4 (4.0)
30-day	16 (16.0)
90-day	73 (73.0)
Additional outcome periods considered in study	8 (8.0)
1-year	2 (2.0)
3-year	4 (4.0)
14-year	2 (2.0)
**Recruitment method**	
Consecutive participants	50 (50.0)
Nonconsecutive sample	1 (1.0)
Unclear or not reported	49 (49.0)
**Number of sites**	
1	41 (41.0)
2	2 (2.0)
3	3 (3.0)
4	0 (0.0)
5–9	13 (13.0)
10+	38 (38.0)
Unclear or not reported	3 (3.0)
**Setting**	
Tertiary	48 (48.0)
Community	10 (10.0)
Tertiary and community	7 (7.0)
Unclear or not reported	35 (35.0)
**Location**	
Urban	50 (50.0)
Rural	0 (0.0)
Urban and rural	8 (8.0)
Unclear or not reported	42 (42.0)
**Study dates were provided**	
Yes	95 (95.0)
**Outcome to be predicted**	
**Same outcome definition (and method of measurement) used in all patients**	
Yes	44 (44.0)
**Explicitly stated that outcome was assessed without knowledge of the candidate predictors (i.e. blinded)**	
Yes	24 (24.0)
**Candidate predictors**	
**All measurement predictors defined with information on how to measure**	
Yes	63 (63.0)
**All measurement predictors defined in terms of when to measure (e.g. pre-hospital, blood pressure measurement time, etc)**	
Yes	47 (47.0)
**Explicitly stated that predictors were assessed blinded for outcome, and for each other**	
Yes	12 (12.0)
**Sample size**	
**Sample size justification provided (practical justification such as using existing study cohort or an RCT data is considered justified)**	
Yes	49 (49.0)
**Flow of participants provided through a flow diagram**	
Yes	21 (21.0)
**Missing data**	
**Number of participants with any missing value (on any of predictors and outcomes) reported**	
Yes	34 (34.0)
**Number of participants with missing data for each predictor reported**	
Yes	28 (28.0)
Number of participants lost to follow-up reported*	
Yes	17 (27.9)
No, unclear or not reported	44 (72.1)
Not applicable (retrospective cohort)	39 (39.0)
Handling of missing data*	
Complete case analysis	14 (15.1)
Predictor with missing values omitted	0 (0.0)
Single imputation	0 (0.0)
Multiple imputation	1 (1.1)
Not handled, unclear, or not reported	78 (83.9)
Not applicable (there were no missing data)	7 (7.0)
**Model performance**	
**Method used for calibration**	
Calibration plot	0 (0.0)
Calibration slope	0 (0.0)
Hosmer-Lemeshow test	5 (5.0)
Unclear or not reported	95 (95.0)
**Method used for discrimination**	
C-statistic/AUC-ROC	79 (79.0)
D-statistic	0 (0.0)
Log-rank	2 (2.0)
Unclear or not reported	19 (19.0)
**Classification measures reported**	
Sensitivity	28 (28.0)
Specificity	28 (28.0)
Predictive values	11 (11.0)
Net reclassification improvement	13 (13.0)
Unclear or not reported	60 (60.0)
**A priori cut points used for the classification measures**	
Yes	25 (25.0)
**Confidence intervals (CIs) provided for the performance measures**	
Yes	65 (65.0)

*Denominator is the applicable cases

#### Introduction (TRIPOD item 3)

Background information includes the medical context and rationale for deriving or validating the model. Background information was provided in 66 (66%) derivation and validation studies.

#### Methods (TRIPOD items 4–12)

Study design or source of data was reported in 99 (99%) of the 100 derivation and validation studies while key study dates (i.e. start and end dates of cohort data collection) were reported in 95 (95%) and study setting (i.e. tertiary, community, or both) was reported in 65 (65%). Fifty-six (56%) recruited participants from multiple sites with 41 (41%) recruiting from one site. Fifty-one (51%) provided information on their recruitment methods of which 50 (50%) recruited consecutive participants. Eligibility criteria for participants was clearly reported in 93 (93%) of the derivation and validation studies.

A clear outcome definition (clinical vs tissue-based) was reported in 80 (80%) of the derivation and validation studies. Forty-four (44%) conveyed that the same outcome definition and method of measurement were used in all patients. The majority, 73 (73%), used a 90-day outcome of stroke followed by a 7-day outcome in 66 (66%) followed by other combinations of outcome time periods. Assessment of the outcome without knowledge of the candidate predictors was reported in 24 (24%). In terms of predictor definitions and measurement, a total of 63 (63%) of the derivation and validation studies reported information on how to measure all measurement predictors while 47 (47%) reported information on when to measure all measurement predictors. Assessment of predictors without knowledge of the outcome or other predictors was reported in 12 (12%).

Sample size justification was reported in 49 (49%) of the derivation and validation studies. Information on missing data and methods of handling the missing data were reported in 15 (16.1%) out of 93 applicable derivations and validations with possible missing data. One (1.1%) study reported that a multiple imputation method was used. Information on risk group creation was provided in 25 (25%).

#### Results (TRIPOD items 13–17)

The flow of participants was reported in 21 (21%) of the derivations and validation studies. Thirty-four (34%) reported information about the prevalence of participants with any missing values, 28 (28%) reported the number of participants with missing data for each predictor, while 7 (7%) reported that there were no missing data.

Measures of discrimination were reported in 81 (81%) of the derivation and validation studies with c-statistic being the most commonly used measure (79%); measures of calibration were reported in 5 (5%), all of which used the Hosmer-Lemeshow test. Sensitivity and specificity were both reported in 28 (28%) while net reclassification improvement and predictive values were reported in 13 (13%) and 11 (11%) respectively.

### Reporting of essential items relevant to all validations

Results of reporting of essential items relevant to validation studies only are presented in Table 3.

**Table 3:** Reporting of essential items applicable to validation studies only

Characteristic	Number (%) ***N*** = 96
**Comparison with derivation cohort**	
**Mention of differences (or no difference) in definitions between the validation and the derivation cohort for each of the following**	
Setting	17 (17.7)
Eligibility criteria	21 (21.9)
Predictors	21 (21.9)
Outcome	21 (21.9)
Unclear or not reported	71 (74.0)
**Mention of differences (or no difference) in definitions between the validation and the derivation cohort**	
Mentioned all four (setting, eligibility, predictors, outcome)	17 (17.7)
Some are mentioned	8 (8.3)
Unclear or not reported	71 (74.0)
**Distribution of important variables (demographics [at least age and sex], predictors, and outcome) presented along with the development study**	
Yes	21 (21.9)
**Model evaluation**	
**Type of external validation**	
Temporal	4 (4.2)
Geographical	64 (66.7)
Methodological/setting (different setting such as ED vs clinic)	26 (27.1)
Unclear or not reported	2 (2.1)
**Model adjusted or updated**	
Yes	23 (24.0)

#### Methods (TRIPOD items 4–12 relevant to validations only)

Among the 96 validation studies, 17 (17.7%) mentioned either similarities or differences in definitions of all four of setting, eligibility criteria, predictors, and outcomes between the validation and derivation of the model. Similarly, the distribution of important variables of at least age and sex was presented along with the development study counterparts for 21 (21.9%) of the validations.

As for model evaluation, the type of external validation (e.g. temporal, geographical, or methodological) was provided for 94 (97.9%) of the validation studies. The majority or 64 (66.7%) of the external validations were geographical. Of the 96 external validations, 23 were external model validations with model updating.

### Reporting of essential items applicable to validations with updating only

Table 4 presents the results of reporting of essential items that are applicable to validation studies with updating only. A rationale for updating the model was provided by all 23 studies. Two (8.7%) studies reported that they have attempted to update the model with less extensive revisions prior to considering more extensive revisions. One study reported applying a method of shrinkage of predictor weights or regression coefficients. When it comes to reporting the results of the updated models, 13 (56.5%) of the studies reported such results (e.g. model specification, model performance, recalibration).

**Table 4:** Reporting of essential items relevant to validation studies with updating only

Characteristic	Number (%) ***N*** = 23
**Rationale provided for updating the model**	
Yes	23 (100.0)
**Authors showed that less extensive update methods were inadequate prior to considering more extensive revisions**^**a**^	
Yes	2 (8.7)
**If the model is derived or updated by methods (c) to (f), was any method of shrinkage of predictor weights or regression coefficients applied**^**b**^	
Uniform shrinkage	0 (0.0)
Penalized estimation	0 (0.0)
Other (method by van Houwelingen)	1 (3.7)
Unclear or not reported	26 (96.3)
**Results from model updating (i.e. model specification, model performance, recalibration) presented (i.e. updated intercept, regression coefficients, discrimination with CI or SE, calibration)**	
Yes	13 (56.5)

^a^*Less extensive updates*: (a) Recalibrating the intercept only, (b) Recalibrating the intercept and adjust the other regression coefficients by a common factor, (c) Category b plus extra adjustment of a subset of the existing coefficients to a different strength, and (d) Category c plus adding new predictors. *Extensive revisions*: (e) Re-estimating all of the original regression coefficients and (f) category e plus adding new additional predictors

^b^The denominator is 27 (*N* = 4 derivations and *N* = 23 external validation with updating)

**Fig. 3:**
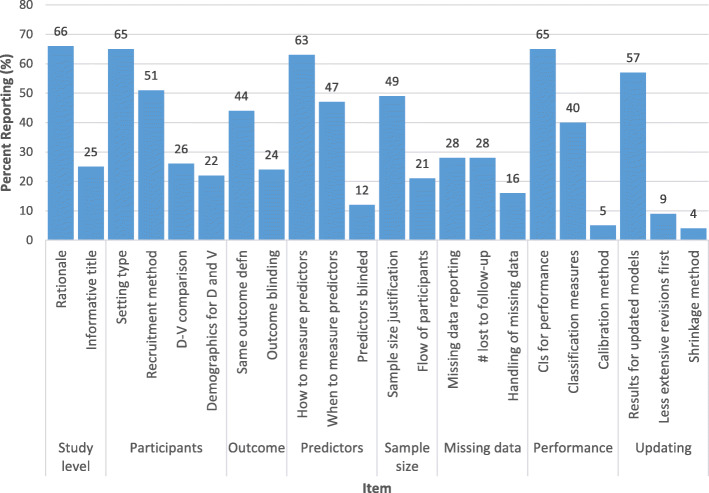
Summary of reporting quality: percentage of studies adhering to each reporting item. D, derivation; V, validation; CIs, confidence intervals

A summary of the quality of reporting can be found in Fig. 3.

## Discussion

### Summary of main findings

We assessed the quality of reporting of derivation and validation studies of prediction models for the prognosis of recurrent stroke in patients with TIA and minor stroke. We found inadequate reporting against selected items in TRIPOD. Items that were especially poorly reported included an informative title, blind assessment of outcome and predictors, sample size justification, use of shrinkage methods, reporting and handling of missing data, reporting of all performance measurements, and comparability between the validation and derivation dataset. Source of data, eligibility criteria, and study setting had better quality of reporting.

Incomplete reporting of blinding is detrimental to assessment of risks of bias and appraisals of the quality of prognostic models. Inadequate sample sizes, a common problem with prediction models, could lead to overfitting and the performance of a prognostic model being overestimated [36, 37]. Sample size justifications for derivation and validation studies are often based on the concept of events per variable (EPV) [10]. However, there is disagreement as to what the EPV should be [10, 38]. More recently, sample size calculation methods have been developed based on the total number of participants, the number of events relative to the number of potential candidate predictor parameters, the outcome proportion (incidence) in the study population, and the expected predictive performance of the model [36]. Missing data can present several problems including reduction in statistical power, bias in the estimation of parameters due to data loss, reduction in representativeness of the samples, and complications in the analysis of the study leading to invalid conclusions such as distorted performance of the prediction model [39]. Failure to adequately report on measures of discrimination and calibration prevents users from making informed decisions about the likely accuracy of the model when used in practice. When validating a model externally, similarities or differences in setting, eligibility criteria, predictors, and outcomes between the validation and derivation dataset need to be reported to understand the extent of reproducibility and generalizability of the model.

As in other systematic reviews, we found that some items are less well reported than others. Although it would be ideal to have all items completely reported, they are not equally important for the appraisal of a prediction model’s performance. However, they are all still important for the likelihood of future validation and uptake in clinical practice. For example, if information on handling of missing data is not reported, we cannot be certain of the true performance of the prediction model; on the other hand, if the title is not adequately reported as recommended in the TRIPOD statement, it does not mean that the quality of the prediction model is compromised, although retrieval of studies for possible updating would be compromised.

### Comparison with other reviews

Our findings are comparable to those in several other systematic reviews of prediction models, published prior to and after the publication of the TRIPOD statement. A systematic review by Heus et al. assessed reporting quality of prediction model studies within 37 clinical domains [20]. Their review excluded studies published prior to the publication of the TRIPOD statement in 2015. Reporting of background information, missing data, and calibration were better than in our review which may be explained by the fact that their review focused on high-impact journals only. Jiang et al. [21] studied the quality of reporting of derivation and validation of melanoma prediction model studies with no restriction on the date of publication. Their findings aligned with our findings although they found better reporting of blinding of predictors and comparability of validation and derivation datasets. A recent systematic review by Najafabadi et al. examined pre- and post-TRIPOD publications in seven high-impact medical journals and found that although there have been some improvements in the methodological conduct, such as better reporting of missing data, use of multiple imputations, reporting the full prediction model and reporting information on performance measures, the overall quality of reporting has not improved [25].

### Strengths and limitations

To our knowledge, this is the first systematic review specifically evaluating the reporting quality of derivation and validation studies of prediction models for the prognosis of stroke in patients with TIA. Our results add to the growing number of studies finding poor reporting quality of prediction models.

Our study has some limitations. Although we did not extract information on all TRIPOD items, we extracted and reported most items with an emphasis on reporting rather than methodological conduct. An item that we missed extracting was the reporting of a full model equation. Although we have attempted to extract information on each item in accordance with the TRIPOD statement, we may have been overly conservative in our assessment of some items: for example, we considered blinding of outcome and predictors as reported if and only if they were explicitly mentioned by the authors.

Although we have searched through two of the major medical databases (Medline and Embase) with the help of two experienced medical librarians and with a sensitive search strategy, we may have missed some eligible articles due to inadequate reporting of information by some authors. In addition, we excluded five studies published in non-English languages due to language barriers. Furthermore, our search covered publications up to February 04, 2020, after which there may be additional publications. However, given that this is a review of the quality of reporting and the comparability of our findings to existing systematic reviews, it is unlikely that any possible missed articles would change the conclusion of this systematic review. A final limitation is that the full-text screening and data extractions were conducted by a single reviewer. However, given the objective nature of many items, it is unlikely that there would have been substantial misclassification.

## Conclusion

Current reporting of multivariable prediction models for the prognosis of stroke in patients with TIA do not meet TRIPOD requirements for reporting. Essential items in need of improvement are providing an informative title, providing a justification for the sample size, providing information on missing data and handling of missing data, blinding of outcome and predictors, applying and reporting a shrinkage method, and clear reporting around comparability between the validation and derivation cohorts when validating. An example prediction model for the prognosis of stroke with a high number of items reported is the validation of the Canadian TIA score study [40]. In addition to adhering to the TRIPOD statement, more comprehensive guidance with breakdown of items by study types and examples with templates would be helpful. In other words, to provide details of what is expected of authors for each item with examples and to provide templates for the authors working on prediction model development studies without external validation, prediction model development studies with external validation, and external model validation studies with or without model updating. In addition, transparent and complete reporting can be facilitated by journals and peer reviewers requiring that authors follow the TRIPOD guidelines when submitting a derivation or validation study. Finally, we found a large number of studies validating and updating existing models, in accordance with recommendations that a prediction model be updated rather than a new one created [35]. However, additional guidance is required with respect to validating and updating a prediction model.

## Supplementary Information

**Additional file 1.** PRISMA checklist.

**Additional file 2 : Table S1.** Key items used to guide the framing of the review aim, search strategy, and study inclusion and exclusion criteria - and - **Table S2.** List of extracted items. This file contains adapted TRIPOD data elements used to design and extract the data.

**Additional file 3.** Literature Search Strategy.

**Additional file 4.** List of included studies.

## Data Availability

Not applicable

## Abbreviations

CHARMS: CHecklist for critical Appraisal and data extraction for systematic Reviews of prediction Modelling Studies
EVP: Events per variable
MeSH: Medical Subject Headings
PRESS: Peer Review of Electronic Search Strategies
PRISMA: Preferred Reporting Items for Systematic Review and Meta-Analysis
PROBAST: Prediction model Risk Of Bias ASsessment Tool
PROSPERO: The International Prospective Register of Systematic Reviews
TIA: Transient ischemic attack
TRIPOD: Transparent Reporting of a Multivariable Prediction Model for Individual Prognosis or Diagnosis
