# Supplementary material for: Quality and transparency of reporting derivation and validation prognostic studies of recurrent stroke in patients with TIA and minor stroke: a systematic review
Source: Diagn Progn Res. 2022 May 19;6:9. doi: 10.1186/s41512-022-00123-z (PMC9118704; doi:10.1186/s41512-022-00123-z)
Supplement: Supplementary file 2 — Additional file 2 : Table S1. Key items used to guide the framing of the review aim, search strategy, and study inclusion and exclusion criteria - and - Table S2. List of extracted items. This file contains adapted TRIPOD data elements used to design and extract the data. [file 41512_2022_123_MOESM2_ESM.docx]

**Table 1**. Key items used to guide the framing of the review aim, search strategy, and study inclusion and exclusion criteria.

| **Item** | **This Review** |
| --- | --- |
| **Prognostic versus diagnostic prediction model** | Prognostic prediction models |
| **Intended scope of the review** | Models to inform physician’s therapeutic decision making |
| **Type of prediction modelling studies** | Prediction model development without external validation  Prediction model development with external validation  External model validation with and without model updating |
| **Target population to whom the prediction model applies** | Patients diagnosed with TIA/Minor Stroke |
| **Outcome to be predicted** | Subsequent Stroke (whether primary or secondary outcome) |
| **Time span of prediction** | Subsequent Stroke within 90 days |
| **Intended moment of using the model** | Models to be used at the moment of diagnosis of TIA/Minor Stroke |

**Table 2**. List of extracted items

| **Study Characteristics** | |
| --- | --- |
| Title | - Reporting in the title of the study as derivation or validation of a multivariable prediction model (or synonyms), the target population, and the outcome to be predicted is reported in the title (Y/N). All the items need to be reported to score a yes. |
| Country of study |  |
| Source and role of funders | - The source of funding is reported or there is explicit mention that there was no external funding involved AND the role of funders is reported when there was funding (Y/N). |
|  |  |
| **Items applicable to both derivation and validation studies*** | |
| Rationale | - Rationale for developing or validating the multivariable prediction model (Y/N). Any sort of rationale is considered. |
| Participants | - Study setting (tertiary, community, or both) - Location (urban, rural, or both) - Recruitment method (consecutive, nonconsecutive participants) - Number of centres - Definition (multi-select) used for inclusion of participants (Time-based TIA, tissue-based TIA, time-based minor stroke, tissue-based minor stroke) |
| Source of data | - Source of data (prospective, retrospective registry, retrospective chart review, retrospective data from previous studies) |
| Study dates | - Study start date and end dates are both provided (Y/N) |
| Outcome | - Definition used for outcome (clinical, tissue-based) - Timing of outcome (2-day, 7-day, 30-day, 90-day, others) - Same outcome definition and method of measurement used in all patients (Y/N). Authors should indicate that an attempt was made to ensure the same definition was used for all patients. For example, for retrospective cohorts it is possible that stroke was defined differently for some of the patients based on medical imaging. It has to be clear that the same definition is used otherwise we flag as unclear (that we have grouped together into a No). - Outcome assessed without knowledge of the candidate predictors (Y/N). For retrospective studies it is possible that the outcome was assessed after information about predictors was reviewed in a chart. Authors need to clearly state that. |
| Candidate predictors | - All measurement predictors defined with information (enough to be able to replicate) on how to measure (Y/N). If no measurement predictors are present we select a yes. If multiple measurement predictors are available, information on all need to be provided in order to score a yes. - All measurement predictors defined with information on when to measure (Y/N). As with above, we score a yes if no measurement predictors were available. - Clear description whether predictor assessments were blinded for outcome and for each other (Y/N) |
| Sample size | - Sample size justification is provided (Y/N). It is explained how the study sample size was arrived at such as statistical grounds or practical/logistical grounds (e.g. an existing study cohort or data set of a RCT) |
| Missing data | - Number of participants with any missing value for the outcome or predictors reported (Y/N) - Number of participants with missing data for each predictor reported (Y/N) - Number of participants lost to follow-up reported in case of prospective cohorts (Y/N) - How missing data was handled (complete case analysis, predictor with missing values omitted, single imputation, multiple imputation, Not handled/unclear or not reported, or not applicable if there were no missing data). If there is no missing data, there should be an explicit mention that there is no missing data for all predictors and outcome, otherwise it will be considered unclear or not reported. |
| Model performance | - Method was used for calibration (calibration plot, calibration slope, Hosmer-Lemeshow test) - Method used for discrimination (C-statistic/AUC, D-statistic, log-rank) - Which classification measures are reported (sensitivity, specificity, predictive values, net reclassification improvement) - Explicitly mentioned that a priori cut-points were used for the classification measures or not (Y/N) - Is the confidence interval (or standard error) for at least the discrimination measure presented? (Y/N) |
|  |  |
| **Items applicable to validation studies only** | |
| Comparisons with development study | - Differences or similarities in definitions with the development study are described. Mentioning of similarities or differences in each of the four (setting, eligibility criteria, predictors and outcome) as a multi-select - Distribution of important demographic variables (at least age and sex), are presented along with the development study (Y/N) |
| External validation type | - The type of external validation (temporal, geographical, methodological/setting (different setting such as ED versus clinic)) |
| Model update | - Was the model updated? (Y/N) |
|  |  |
| **Items applicable to model updating studies only** | |
| Rationale for update | - Is a clear rationale provided for updating the model (Y/N)? Any rationale for trying to come up with a ‘better’ prediction model is accepted |
| Priority of update | - Have the authors shown that less extensive updating methods were inadequate prior to considering more extensive revisions? (Y/N).   *Less extensive updates include*: a) Recalibrating the intercept only, b) Recalibrating the intercept and adjust the other regression coefficients by a common factor, c) Category b plus extra adjustment of a subset of the existing coefficients to a different strength, and d) Category c plus adding new predictors}.  *Extensive revisions include*: e) Re-estimating all of the original regression coefficients, and f) category e plus adding new additional predictors |
| Shrinkage method | - If the model is derived or updated by methods (c) to (f) above, method of shrinkage of predictor weights or regression coefficients applied (No shrinkage, uniform shrinkage, penalized estimation, other) |
| Results of update | - If the model is updated, are the results from model updating (i.e., model specification, model performance, and recalibration) presented? (i.e. updated intercept, regression coefficients, discrimination with CI or SE, calibration) (Y/N). A minimum of discrimination is required to answer Yes. |

Y, Yes; N, No.

*For Y/N items: No, unclear or not reported were combined
