# Supplementary material for: Quality and transparency of reporting derivation and validation prognostic studies of recurrent stroke in patients with TIA and minor stroke: a systematic review
Source: Diagn Progn Res. 2022 May 19;6:9. doi: 10.1186/s41512-022-00123-z (PMC9118704; doi:10.1186/s41512-022-00123-z)
Supplement: Supplementary file 3 — Additional file 3. Literature Search Strategy. [file 41512_2022_123_MOESM3_ESM.docx]

**Literature Search Strategy**

Medline:

1     exp Stroke/
2     stroke.tw.
3     (cerebrovascular adj1 accident*).tw.
4     (cerebral adj1 vascular adj1 accident*).tw.
5     (cva or cvas).tw.
6     (cerebral adj1 accident*).tw.
7     (brain adj1 isch?emia).tw.
8     (cerebral adj1 isch?emia).tw.
9     (cerebrovascular adj1 isch?emia).tw.
10     (cerebral adj1 vascular adj1 isch?emia).tw.
11     (cerebral adj1 infarction).tw.
12     (brain adj1 infarction).tw.
13     (brain adj1 stem adj1 infarction).tw.
14     (cerebrovascular adj1 apoplex*).tw.
15     (brain adj1 vascular adj1 accident*).tw.
16     1 or 2 or 3 or 4 or 5 or 6 or 7 or 8 or 9 or 10 or 11 or 12 or 13 or 14 or 15
17     Ischemic Attack, Transient/
18     (tia or tias).tw.
19     (transient adj1 isch?emic adj1 attack*).tw.
20     (transient adj1 brain adj1 attack*).tw.
21     (transient adj1 cerebral adj1 attack*).tw.
22     (transient adj1 cerebral adj1 isch?em*).tw.
23     (transient adj1 brain adj1 stem adj1 isch?em*).tw.
24     (transient adj1 brainstem adj1 isch?em*).tw.
25     17 or 18 or 19 or 20 or 21 or 22 or 23 or 24
26     decision support techniques/
27     Prognosis/mt [Methods]
28     Nomograms/
29     nomogram*.tw.
30     (predict* adj2 model*).tw.
31     (predict* adj2 rule*).tw.
32     (predict* adj2 score*).tw.
33     (decision adj2 model*).tw.
34     (decision adj2 rule*).tw.
35     (decision adj2 score*).tw.
36     (risk adj2 model*).tw.
37     (risk adj2 rule*).tw.
38     (risk adj2 score*).tw.
39     (decision adj2 aid).tw.
40     validation.tw.
41     26 or 27 or 28 or 29 or 30 or 31 or 32 or 33 or 34 or 35 or 36 or 37 or 38 or 39 or 40
42     16 and 25 and 41

Embase:

1 exp cerebrovascular accident/

2 stroke*.tw.

3 (cerebrovascular adj1 accident*).tw.

4 (cerebral adj1 vascular adj1 accident*).tw.

5 (cva or cvas).tw.

6 (cerebral adj1 accident*).tw.

7 (brain adj1 isch?em*).tw.

8 (cerebral adj1 isch?em*).tw.

9 (cerebrovascular adj1 isch?em*).tw.

10 (cerebral adj1 vascular adj1 isch?em*).tw.

11 (cerebral adj1 infarction*).tw.

12 (brain adj1 infarction*).tw.

13 (brain adj1 stem adj1 infarction*).tw.

14 (cerebrovascular adj1 apoplex*).tw.

15 (brain adj1 vascular adj1 accident*).tw.

16 1 or 2 or 3 or 4 or 5 or 6 or 7 or 8 or 9 or 10 or 11 or 12 or 13 or 14 or 15

17 transient ischemic attack/

18 (tia or tias).tw.

19 (transient adj1 isch?emic adj1 attack*).tw.

20 (transient adj1 brain adj1 attack*).tw.

21 (transient adj1 cerebral adj1 attack*).tw.

22 (transient adj1 cerebral adj1 isch?em*).tw.

23 (transient adj1 brain adj1 stem adj1 isch?em*).tw.

24 (transient adj1 brainstem adj1 isch?em*).tw.

25 17 or 18 or 19 or 20 or 21 or 22 or 23 or 24

26 decision support system/

27 prognosis/

28 nomogram/

29 nomogram*.tw.

30 ((predicti* or decision? or prognos* or risk? or derivation? or derive? or develop* or validation? or validate? or refinement? or refine? or computer or web-based) adj5 (model? or rule? or scori* or score? or tool? or index or indexes or indices or scale? or algorithm? or nomogram? or aid? or aided or support or system? or criteria or stratif* or identification or calculator? or instrument?)).tw.

31 ((scori* or score?) adj5 (model? or rule? or tool? or index or indexes or indices or scale? or algorithm? or nomogram? or aid? or aided or support or system? or criteria or stratif* or identification or calculator? or instrument?)).tw.

32 validation.tw.

33 algorithm.tw.

34 26 or 27 or 28 or 29 or 30 or 31 or 32 or 33

35 16 and 25 and 34
