# Supplementary material for: Quality and transparency of reporting derivation and validation prognostic studies of recurrent stroke in patients with TIA and minor stroke: a systematic review
Source: Diagn Progn Res. 2022 May 19;6:9. doi: 10.1186/s41512-022-00123-z (PMC9118704; doi:10.1186/s41512-022-00123-z)
Supplement: Supplementary file 4 — Additional file 4. List of included studies. [file 41512_2022_123_MOESM4_ESM.docx]

**List of included studies**

1. Johnston SC, Hamilton-Bruce MA, Price C, et al. Short-term Prognosis After Emergency Department Diagnosis of TIA. *JAMA*. 2000;284(22):2901. doi:10.1001/jama.284.22.2901

2. Rothwell P, Giles M, Flossmann E, et al. A simple score (ABCD) to identify individuals at high early risk of stroke after transient ischaemic attack. *Lancet*. 2005;366(9479):29-36. doi:10.1016/S0140-6736(05)66702-5

3. Cucchiara BL, Messe SR, Taylor RA, et al. Is the ABCD score useful for risk stratification of patients with acute transient ischemic attack? *Stroke*. 2006;37(7):1710-1714. doi:10.1161/01.STR.0000227195.46336.93

4. Tsivgoulis G, Spengos K, Manta P, et al. Validation of the ABCD score in identifying individuals at high early risk of stroke after a transient ischemic attack: A hospital-based case series study. *Stroke*. 2006;37(12):2892-2897. doi:10.1161/01.STR.0000249007.12256.4a

5. Koton S, Rothwell PM. Performance of the ABCD and ABCD2 scores in TIA patients with carotid stenosis and atrial fibrillation. *Cerebrovasc Dis*. 2007;24(2-3):231-235. doi:10.1159/000104483

6. Bray JE, Coughlan K, Bladin C. Can the ABCD Score be dichotomised to identify high-risk patients with transient ischaemic attack in the emergency department? *Emerg Med J*. 2007;24(2):92-95. doi:10.1136/emj.2006.041624

7. Johnston SC, Rothwell PM, Nguyen-Huynh MN, et al. Validation and refinement of scores to predict very early stroke risk after transient ischaemic attack. *Lancet*. 2007;369(9558):283-292. doi:10.1016/S0140-6736(07)60150-0

8. Selvarajah JR, Smith CJ, Hulme S, et al. Prognosis in patients with transient ischaemic attack (TIA) and minor stroke attending TIA services in the North West of England: The NORTHSTAR Study. *J Neurol Neurosurg Psychiatry*. 2008;79(1):38-43. doi:10.1136/jnnp.2007.129163

9. Sciolla R, Melis F. Rapid identification of high-risk transient ischemic attacks: Prospective validation of the ABCD score. *Stroke*. 2008;39(2):297-302. doi:10.1161/STROKEAHA.107.496612

10. Fothergill A, Christianson TJH, Brown RD, Rabinstein AA. Validation and refinement of the ABCD2 score: A population-based analysis. *Stroke*. 2009;40(8):2669-2673. doi:10.1161/STROKEAHA.109.553446

11. Ay H, Arsava EM, Johnston SC, et al. Clinical- and imaging-based prediction of stroke risk after transient ischemic attack: The CIP model. *Stroke*. 2009;40(1):181-186. doi:10.1161/STROKEAHA.108.521476

12. Cucchiara BL, Messe SR, Sansing L, et al. D-dimer, Magnetic Resonance Imaging Diffusion-weighted Imaging, and ABCD2 Score for Transient Ischemic Attack Risk Stratification. *J Stroke Cerebrovasc Dis*. 2009;18(5):367-373. doi:10.1016/j.jstrokecerebrovasdis.2009.01.006

13. Tsivgoulis G, Stamboulis E, Sharma VK, et al. Multicenter external validation of the ABCD2 score in triaging TIA patients. *Neurology*. 2010;74(17):1351-1357. doi:10.1212/WNL.0b013e3181dad63e

14. Sheehan OC, Kyne L, Kelly LA, et al. Population-based study of ABCD2 score, carotid stenosis, and atrial fibrillation for early stroke prediction after transient ischemic attack: The North Dublin TIA study. *Stroke*. 2010;41(5):844-850. doi:10.1161/STROKEAHA.109.571844

15. Ong MEH, Chan YH, Lin WP, Chung WL. Validating the ABCD2 Score for predicting stroke risk after transient ischemic attack in the ED. *Am J Emerg Med*. 2010;28(1):44-48. doi:10.1016/j.ajem.2008.09.027

16. Nguyen H, Kerr D, Kelly AM. Comparison of prognostic performance of scores to predict risk of stroke in ED patients with transient ischaemic attack. *Eur J Emerg Med*. 2010;17(6):346-348. doi:10.1097/MEJ.0b013e328337b1c6

17. Holzer K, Feurer R, Sadikovic S, et al. Prognostic value of the ABCD2score beyond short-term follow-up after transient ischemic attack (TIA) - A cohort study. *BMC Neurol*. 2010;10. doi:10.1186/1471-2377-10-50

18. Harrison JK, Sloan B, Dawson J, Lees KR, Morrison DS. The ABCD and ABCD2 as predictors of stroke in transient ischemic attack clinic outpatients: A retrospective cohort study over 14 years. *Qjm*. 2010;103(9):679-685. doi:10.1093/qjmed/hcq108

19. Asimos AW, Johnson AM, Rosamond WD, et al. A Multicenter Evaluation of the ABCD2 Score’s Accuracy for Predicting Early Ischemic Stroke in Admitted Patients With Transient Ischemic Attack. *Ann Emerg Med*. 2010;55(2):201-210.e5. doi:10.1016/j.annemergmed.2009.05.002

20. Giles MF, Albers GW, Amarenco P, et al. Addition of brain infarction to the ABCD2 score (ABCD 2I): A collaborative analysis of unpublished data on 4574 patients. *Stroke*. 2010;41(9):1907-1913. doi:10.1161/STROKEAHA.110.578971

21. Merwick Á, Albers GW, Amarenco P, et al. Addition of brain and carotid imaging to the ABCD2 score to identify patients at early risk of stroke after transient ischaemic attack: A multicentre observational study. *Lancet Neurol*. 2010;9(11):1060-1069. doi:10.1016/S1474-4422(10)70240-4

22. Stead LG, Suravaram S, Bellolio MF, et al. An assessment of the incremental value of the ABCD2 score in the emergency department evaluation of transient ischemic attack. *Ann Emerg Med*. 2011;57(1):46-51. doi:10.1016/j.annemergmed.2010.07.001

23. Sanders LM, Srikanth VK, Psihogios H, Wong KK, Ramsay D, Phan TG. Clinical predictive value of the ABCD2 score for early risk of stroke in patients who have had transient ischaemic attack and who present to an Australian tertiary hospital. *Med J Aust*. 2011;194(3):135-138. doi:10.5694/j.1326-5377.2011.tb04196.x

24. Perry JJ, Sharma M, Sivilotti MLA, et al. Prospective validation of the ABCD2 score for patients in the emergency department with transient ischemic attack. *CMAJ*. 2011;183(10):1137-1145. doi:10.1503/cmaj.101668

25. Meng X, Wang Y, Liu L, et al. Validation of the ABCD 2-I score to predict stroke risk after transient ischemic attack. *Neurol Res*. 2011;33(5):482-486. doi:10.1179/016164111X13007856084043

26. Giles MF, Albers GW, Amarenco P, et al. Early stroke risk and ABCD2 score performance in tissue- Vs time-defined TIA: A multicenter study. *Neurology*. 2011;77(13):1222-1228. doi:10.1212/WNL.0b013e3182309f91

27. Cancelli I, Janes F, Gigli GL, et al. Incidence of transient ischemic attack and early stroke risk: Validation of the ABCD2 score in an Italian population-based study. *Stroke*. 2011;42(10):2751-2757. doi:10.1161/STROKEAHA.110.612705

28. Katan M, Nigro N, Fluri F, et al. Stress hormones predict cerebrovascular re-events after transient ischemic attacks. *Neurology*. 2011;76(6):563-566. doi:10.1212/WNL.0b013e31820b75e6

29. Arsava EM, Furie KL, Schwamm LH, Sorensen AG, Ay H. Prediction of early stroke risk in transient symptoms with infarction: Relevance to the new tissue-based definition. *Stroke*. 2011;42(8):2186-2190. doi:10.1161/STROKEAHA.110.604280

30. Purroy F, Caballero PEJ, Gorospe A, et al. Prediction of early stroke recurrence in transient ischemic attack patients from the PROMAPA study: A comparison of prognostic risk scores. *Cerebrovasc Dis*. 2012;33(2):182-189. doi:10.1159/000334771

31. Ghia D, Thomas P, Cordato D, et al. Low positive predictive value of the ABCD2 score in emergency department transient ischaemic attack diagnoses: The South Western Sydney Transient Ischaemic Attack Study. *Intern Med J*. 2012;42(8):913-918. doi:10.1111/j.1445-5994.2011.02564.x

32. Ghandehari K, Ahmadi F, Ebrahimzadeh S, Shariatinezhad K, Ghandehari K. Assessment of ABCD2 scale in patients with transient ischaemic attack or stroke. *Neurol Neurochir Pol*. 2012;46(5):421-427. doi:10.5114/ninp.2012.31351

33. Ghandehari K, Ahmadi F, Ebrahimzadeh S, Shariatinezhad K, Ghandehari K. The ABCD2 Score is Highly Predictive of Stroke in Minor Ischemic Stroke Patients. *Transl Stroke Res*. 2012;3(2):273-278. doi:10.1007/s12975-012-0146-0

34. Engelter ST, Amort M, Jax F, et al. Optimizing the risk estimation after a transient ischaemic attack - The ABCDE⊕ score. *Eur J Neurol*. 2012;19(1):55-61. doi:10.1111/j.1468-1331.2011.03428.x

35. Song B, Fang H, Zhao L, et al. Validation of the ABCD3-I score to predict stroke risk after transient ischemic attack. *Stroke*. 2013;44(5):1244-1248. doi:10.1161/STROKEAHA.113.000969

36. Purroy F, Jiménez-Caballero PE, Mauri-Capdevila G, et al. Predictive value of brain and vascular imaging including intracranial vessels in transient ischaemic attack patients: External validation of the ABCD3-I score. *Eur J Neurol*. 2013;20(7):1088-1093. doi:10.1111/ene.12141

37. Ozpolat C, Denizbasi A, Akoglu H, Onur O, Emre Eroglu S, Demir H. Use of ABCD2 risk scoring system to determine the short-term stroke risk in patients presenting to emergency department with transient ischaemic attack. *J Pak Med Assoc*. 2013;63(9):1142-1146.

38. Chardoli M, Khajavi A, Nouri M, Rahimi-Movaghar V. Value of ABCD2 in predicting early ischemic stroke in patients diagnosed with transient ischemic attack. *Acta Med Iran*. 2013;51(9):611-614. doi:10.5249/jivr.v4i3.408

39. Perry JJ, Sharma M, Sivilotti MLA, et al. A prospective cohort study of patients with transient ischemic attack to identify high-risk clinical characteristics. *Stroke*. 2014;45(1):92-100. doi:10.1161/STROKEAHA.113.003085

40. Kiyohara T, Kamouchi M, Kumai Y, et al. ABCD3 and ABCD3-I scores are superior to ABCD2 score in the prediction of short- and long-term risks of stroke after transient ischemic attack. *Stroke*. 2014;45(2):418-425. doi:10.1161/STROKEAHA.113.003077

41. Johansson E, Bjellerup J, Wester P. Prediction of recurrent stroke with ABCD2 and ABCD3 scores in patients with symptomatic 50-99% carotid stenosis. *BMC Neurol*. 2014;14(1):1-7. doi:10.1186/s12883-014-0223-y

42. Galvin R, Atanassova PA, Motterlini N, Fahey T, Dimitrov BD. Long-term risk of stroke after transient ischaemic attack: A hospital-based validation of the ABCD2 rule. *BMC Res Notes*. 2014;7(1):1-7. doi:10.1186/1756-0500-7-281

43. Chiu LHS, Yau WH, Leung LP, et al. Short-Term Prognosis of Transient Ischemic Attack and Predictive Value of the ABCD2 Score in Hong Kong Chinese. *Cerebrovasc Dis Extra*. 2014;4(1):40-51. doi:10.1159/000360074

44. De Marchis GM, Weck A, Audebert H, et al. Copeptin for the prediction of recurrent cerebrovascular events after transient ischemic attack: Results from the CoRisk study. *Stroke*. 2014;45(10):2918-2923. doi:10.1161/STROKEAHA.114.005584

45. Wang J, Wu J, Liu R, Gao F, Hu H, Yin X. The ABCD score is better for stroke risk prediction after anterior circulation TIA compared to posterior circulation TIA. *Int J Neurosci*. 2015;125(1):50-55. doi:10.3109/00207454.2014.905777

46. Dai Q, Sun W, Xiong Y, et al. From clinical to tissue-based dual TIA: Validation and refinement of ABCD3-I score. *Neurology*. 2015;84(14):1426-1432. doi:10.1212/wnl.0000000000001444

47. Ottaviani M, Vanni S, Moroni F, Peiman N, Boddi M, Grifoni S. Urgent carotid duplex and head computed tomography versus ABCD2 score for risk stratification of patients with transient ischemic attack. *Eur J Emerg Med*. 2016;23(1):19-23. doi:10.1097/MEJ.0000000000000165

48. Knoflach M, Lang W, Seyfang L, et al. Predictive value of ABCD2 and ABCD3-I scores in TIA and minor stroke in the stroke unit setting. *Neurology*. 2016;87(9):861-869. doi:10.1212/WNL.0000000000003033

49. Dutta D, Bailey SJ. Validation of ABCD2 scores ascertained by referring clinicians: A retrospective transient ischaemic attack clinic cohort study. *Emerg Med J*. 2016;33(8):543-547. doi:10.1136/emermed-2015-205519

50. Almasi M, Hodjati Firoozabadi N, Ghasemi F, Chardoli M. The Value of ABCD2F Scoring System (ABCD2 Combined with Atrial Fibrillation) to Predict 90-Day Recurrent Brain Stroke. *Neurol Res Int*. 2016;2016. doi:10.1155/2016/8191659

51. Chardoli M, Firoozabadi NH, Nouri M, Rahimi-Movaghar V. Value of ABCD2-F in predicting cerebral ischemic attacks: Three months follow-up after the primary attack. *Acta Med Iran*. 2016;54(6):391-394.

52. Appelros P, Berglund MH, Ström JO. Long-term risk of stroke after transient ischemic attack. *Cerebrovasc Dis*. 2017;43(1-2):25-30. doi:10.1159/000451061

53. Vigen T, Thommessen B, Rønning OM. Stroke Risk Is Low after Urgently Treated Transient Ischemic Attack. *J Stroke Cerebrovasc Dis*. 2018;27(2):291-295. doi:10.1016/j.jstrokecerebrovasdis.2017.08.037

54. Mayer L, Ferrari J, Krebs S, et al. ABCD3-I score and the risk of early or 3-month stroke recurrence in tissue- and time-based definitions of TIA and minor stroke. *J Neurol*. 2018;265(3):530-534. doi:10.1007/s00415-017-8720-8

55. Huan Y, Chaoyang Z, Kai D, Chunhua S, Xin Z, Yue Z. Predictive value of head-neck CTA combined with ABCD2 scale score for patients with cerebral infarction of vertebrobasilar transient ischemic attack (TIA). *Med Sci Monit*. 2018;24:9001-9006. doi:10.12659/MSM.909470

56. Ildstad F, Ellekjær H, Wethal T, et al. Stroke risk after transient ischemic attack in a Norwegian prospective cohort. *BMC Neurol*. 2019;19(1):1-7. doi:10.1186/s12883-018-1225-y

57. Xi HY, Si ZH, Li JC, Zhu JG, Yan HY. Assessment of cerebral infarction after transient cerebral ischemic attack by ABCD2 score combined with the position of intracranial vascular stenosis. *Medicine (Baltimore)*. 2019;98(15):e15081. doi:10.1097/MD.0000000000015081

58. Dahlquist RT, Young JM, Reyner K, et al. Initiation of the ABCD3-I algorithm for expediated evaluation of transient ischemic attack patients in an emergency department. *Am J Emerg Med*. 2020;38(4):741-745. doi:10.1016/j.ajem.2019.06.018

59. Zhang C, Zang Y, Hu L, et al. Study on the risk prediction for cerebral infarction after transient ischemic attack: A STROBE compliant study. *Medicine (Baltimore)*. 2020;99(11):e19460. doi:10.1097/MD.0000000000019460

60. Kelly PJ, Camps-Renom P, Giannotti N, et al. A risk score including carotid plaque inflammation and stenosis severity improves identification of recurrent stroke. *Stroke*. 2020:838-845. doi:10.1161/STROKEAHA.119.027268
